# The structure of microbial communities of activated sludge of large-scale wastewater treatment plants in the city of Moscow

**DOI:** 10.1038/s41598-022-07132-4

**Published:** 2022-03-02

**Authors:** Shahjahon Begmatov, Alexander G. Dorofeev, Vitaly V. Kadnikov, Alexey V. Beletsky, Nikolai V. Pimenov, Nikolai V. Ravin, Andrey V. Mardanov

**Affiliations:** 1 Institute of Bioengineering, Research Center of Biotechnology of the Russian Academy of Sciences, Leninsky Prosp, bld. 33-2, Moscow, Russia 119071; 2 Winogradsky Institute of Microbiology, Research Center of Biotechnology of the Russian Academy of Sciences, Leninsky Prosp, bld. 33‑2, Moscow, Russia 119071

**Keywords:** Environmental biotechnology, Microbial communities, Microbial ecology, Microbiome, Water microbiology, Environmental microbiology

## Abstract

Microbial communities in wastewater treatment plants (WWTPs) play a key role in water purification. Microbial communities of activated sludge (AS) vary extensively based on plant operating technology, influent characteristics and WWTP capacity. In this study we performed 16S rRNA gene profiling of AS at nine large-scale WWTPs responsible for the treatment of municipal sewage from the city of Moscow, Russia. Two plants employed conventional aerobic process, one plant—nitrification/denitrification technology, and six plants were operated with the University of Cape Town (UCT) anaerobic/anoxic/oxic process. Microbial communities were impacted by the technology and dominated by the *Proteobacteria*, *Bacteroidota* and *Actinobacteriota*. WWTPs employing the UCT process enabled efficient removal of not only organic matter, but also nitrogen and phosphorus, consistently with the high content of ammonia-oxidizing *Nitrosomonas* sp. and phosphate-accumulating bacteria. The latter group was represented by *Candidatus* Accumulibacter, *Tetrasphaera* sp. and denitrifiers. Co-occurrence network analysis provided information on key hub microorganisms in AS, which may be targeted for manipulating the AS stability and performance. Comparison of AS communities from WWTPs in Moscow and worldwide revealed that Moscow samples clustered together indicating that influent characteristics, related to social, cultural and environmental factors, could be more important than a plant operating technology.

## Introduction

The removal of numerous pollutants produced by agriculture, industry, and households is important for the protection of natural ecosystems and human health. Wastewater treatment plants (WWTPs) employ a series of mechanical and biological processes that convert contaminated water into a sufficiently clean state through a series of steps removing different types of organic and inorganic pollutants^1,2^. Typically, wastewater treatment in large facilities takes place in three stages. The first stage includes physical methods of water purification, the second stage—chemical and/or biological treatment in bioreactors with suspended or attached activated sludge (AS). The third stage is the final treatment of water and its disinfection.

At the second stage, a consortium of microorganisms of AS transforms pollutants into harmless products or into products less hazardous to the environment and humans than the original components^3^. AS is a taxonomically and metabolically diverse microbial community with complex trophic relationships between its members^4^. It is the largest managed artificial ecosystem, continuously functioning in many cases for decades. The composition of the microbial community, which is shaped by both operating conditions and influent characteristics^5,6^, determines the main biochemical processes of wastewater treatment, and its change, for example, the massive development of filamentous forms of bacteria, can lead to a decrease in the efficiency of treatment and the occurrence of emergency situations^7^.

In municipal wastewater treatment plants, microbial consortia of AS are often developed under similar conditions, since the content of the main components of wastewater is limited to a rather narrow range of concentrations (except in some extreme cases of highly diluted or concentrated wastewater), pH is usually 7–8, temperatures vary from 10 to 30 °C^8^. In addition, in modern technologies certain biologically determined rules are followed: maintaining the minimum aerobic age of sludge necessary for the development of nitrification (the retention time of solid matter), ensuring the optimal retention time of the sludge in the anaerobic zone for effective enhanced biological phosphorus removal, optimizing the ratio of biochemical oxygen demand, nitrogen and phosphorus, etc.^3^. Therefore, it can be expected that the composition of microbial communities of activated sludge will contain a common component, which was confirmed by the results of studies comparing the composition of AS communities of various WWTPs^6,9,10^. At the same time a high diversity and differences of microbial communities of AS were noted, which was associated with climatic factors and the specificity of certain treatment plants: the share of the industrial component in the total influent, the temperature regime, the peculiarity of the used technologies and the exploitation of plants^9^. It has been shown that there is a relationship between the diversity and composition of the microbial community and the performance of treatment facilities^2^, although the authors noted that the real effect is not the performance itself, but the variation of indicators such as chemical oxygen demand, the retention time of suspended matter etc.

Despite the extensive application of traditional and modern molecular methods for studying microbial consortia of AS, their ecophysiology, population dynamics and diversity are far from being comprehensively understood. Most microorganisms of activated sludge are not cultivated, and the role of many typical inhabitants is not clearly known^11,12^.

Recently, using a systematic worldwide sampling, a Global Water Microbiome Consortium (GWMC) analysed the 16S ribosomal RNA gene sequences from ~ 1,200 AS samples taken from 269 WWTPs^10^. This study revealed that although the global AS bacterial communities contain ~ 1 billion phylotypes, ASs has a small, global core bacterial community of 28 phylotypes that is strongly linked to WWTP performance^10^. This study showed that although the type of treatment process exerted significant effects on microbial community structures, it was overwhelmed by geographical separation, and the compositions of AS microbial communities were significantly different between any two continents^10^. Although the GWMC study included WWTPs from 23 countries on 6 continents, the distribution of samples was geographically biased and covered mostly North America, Western and Central Europe, Eastern Asia (mostly China), Australasia, and several cities in South America and South Africa^10^.

Another large-scale initiative, the MiDAS project, analyzed samples (mostly AS) from 740 WWTPs using different types of treatment technologies, and represents the largest global sampling of WWTPs to date^13,14^. The resulting full-length 16S rRNA gene reference database, MiDAS 4, represent a comprehensive catalogue of bacteria in wastewater treatment systems and taxonomy from the domain to species level^14^. Although this study targeted WWTPs located in 425 cities, 31 countries on 6 continents, like the GWMC project, MiDAS mostly covered the same geographic regions.

In order to expand the geographical coverage and our knowledge about microbiomes of AS in general, we analyzed the composition of microbial communities of AS at large-scale WWTPs in the city of Moscow (Russia). Although the Moscow WWTPs are among the largest in the world, they (as well as other WWTPs from Russia) were not studied in the framework of GWMC and MiDAS projects. There are only a few studies on the analysis of the composition of microbial communities of AS from Moscow WWTPs using modern high throughput molecular genetic methods. Kallistova et al*.*^15^ using FISH method analyzed AS samples from four Moscow WWTPs and characterized the abundance of the major technologically important microbial groups (ammonium- and nitrite-oxidizing, phosphate-accumulating, foam-inducing, anammox bacteria, and methanogens) in the aeration tanks. Later Shchegolkova et al*.*^16^ performed 16S rRNA gene profiling of AS communities in three WWTPs responsible for processing sewage with different origins: municipal wastewater, slaughterhouse wastewater, and refinery sewage. The taxonomic structures of AS microbiomes were found to become stable in time, and each WWTP demonstrated a distinct pattern^16^. Several studies were devoted to 16S rRNA profiling, metagenomics and FISH studies of nitritation/ anammox wastewater treatment bioreactors applied for the treatment of NH_4_-rich wastewater^17–20^.

In this study, we present the results of an analysis of the composition of AS microbial communities at nine large-scale WWTPs in Moscow employing three different technologies.

## Methods

### Characteristics of WWTPs in the city of Moscow

Wastewater treatment plants of JSC "Mosvodokanal" carry out the treatment of sewage in the city of Moscow. Household and industrial wastewater entering the city sewerage system undergoes a full purification process, including biological treatment with AS. The largest treatment facilities, the Lyuberetskiy and Kuryanovskiy WWTP complexes (hereafter referred to as LOS and KOS, respectively), each with a capacity of about 2 million m^3^ per day, consist of several wastewater treatment units in which a number of modern technologies for biological wastewater treatment are implemented, including biological nutrient removal (carbon, nitrogen, phosphorus)^21–24^. These treatment units (hereafter referred to as WWTPs) at each WWTP complex are fed by the same inflow water but otherwise are independent installations between which there is no transfer and mixing of AS.

After the primary treatment, the wastewater is subjected to purification in bioreactors with suspended activated sludge. The characteristics of the wastewater entering bioreactors at the two WWTP complexes are somehow different. The waters at the Kuryanovskiy WWTP complex contain approximately 1.5 times lower concentrations of organic matter and phosphorus compared to the Lyuberetskiy WWTPs, while the ammonium content do not differ significantly (Table 1).

**Table 1:** Characteristics of analyzed WWTPs.

WWTP ID	WWTP1	WWTP2	WWTP3	WWTP4	WWTP5	WWTP6	WWTP7	WWTP8	WWTP9
WWTPs complex	LOS	LOS	LOS	LOS	LOS	LOS	KOS	KOS	KOS
Technology	SF	N-DN	UCT	UCT	UCT	UCT	CAS	UCT	UCT
WWTP capacity (10^3^ m^3^/day)	1000	500	80	80	80	500	1000	600	600
Bioreactors hydraulic regime	Plug-flow	Plug-flow	Carrousel	Plug-flow	Carrousel	Carrousel	Plug-flow	Plug-flow	Plug-flow
Hydraulic retention time (h)	12	12	9	9	9	12	8	10	10
BOD –inflow (mg/L)	180–190	180–190	180–190	180–190	180–190	170–190	90–130	80–120	90–130
BOD –outflow (mg/L)	3–4	2–3	< 3	< 3	< 3	1–2	5–6	1–3	1–3
COD –inflow (mg/L)	500–550	500–550	500–550	500–550	500–550	450–500	310–350	300–340	310–350
COD –outflow (mg/L)`	30–40	30–40	nd	nd	nd	30–40	30–40	30–40	30–40
N-NH_4_- inflow (mg/L)	40–50	40–55	40–50	40–50	40–50	35–45	40–50	35–45	40–50
N-NH_4_- outflow (mg/L)	5–6	0.4–0,5	< 0,4	< 0,4	< 0,4	0,3–0,4	12–16	0,3–0,5	0,3–0,5
N-NO_3_ – outflow (mg/L)	10–11	10–11	< 9	< 9	< 9	7–8	5–9	8–9	8–9
P-PO_4_ – inflow (mg/L)	4–5	4–5	4–5	4–5	4–5	4–5	2–4	2–4	2–4
P-PO_4_ – outflow (mg/L)	2,5–3,5	3–4	< 1	< 1	< 1	0,2–0,3	0,3–0,6	0,2–0,4	0,2–0,4

**Figure 1:**
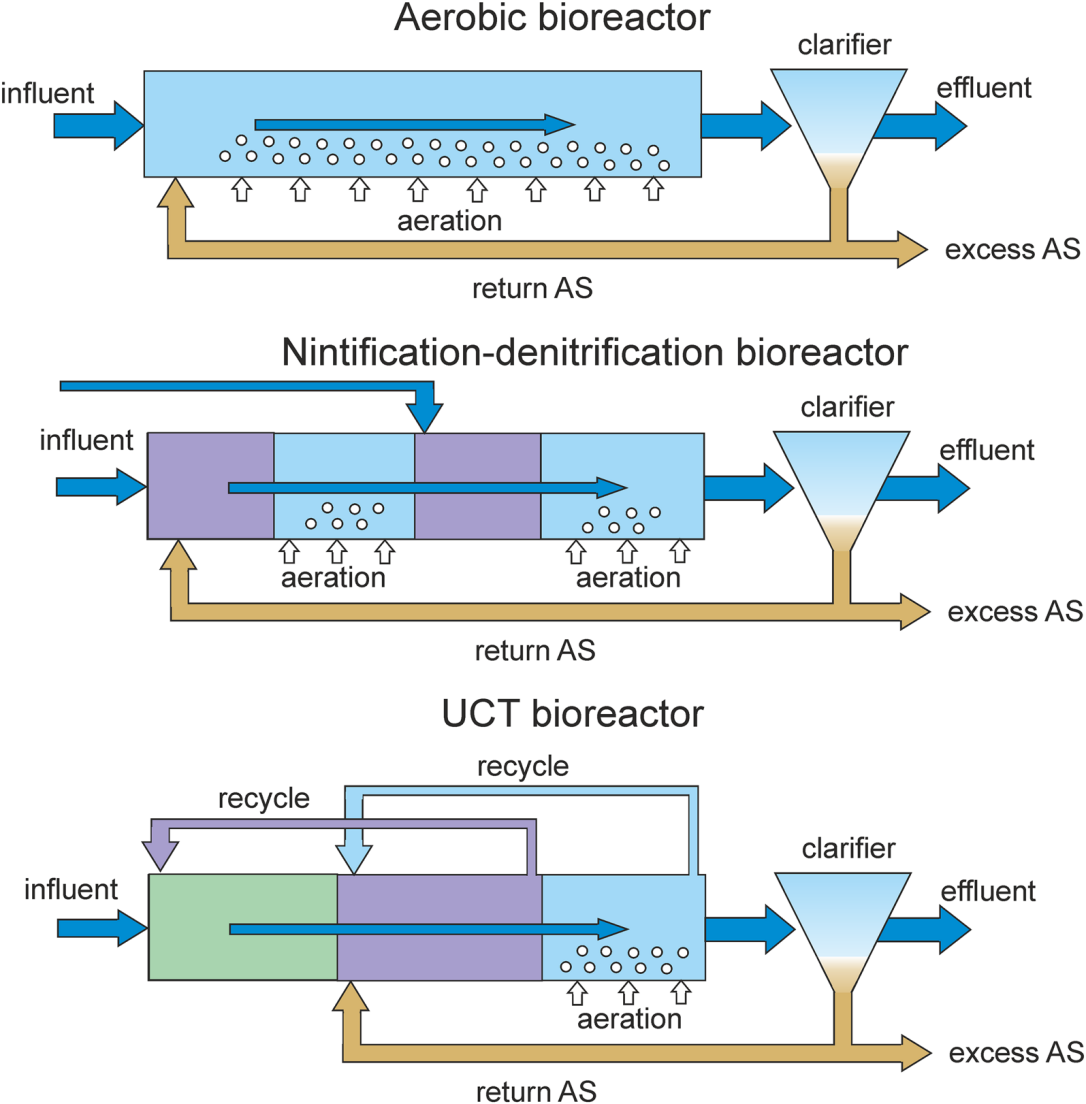
Schemes of three types of bioreactors showing the different compartments and flow directions. Anaerobic, anoxic and oxic zones are colored in green, violet and blue, respectively.

All bioreactors are continuous: the inflowing wastewater and the returned AS are continuously fed to the inlet (or to a certain point) of the bioreactor, where they are mixed. Then the mixed liquor passes the bioreactor and enters the clarifier where the sludge is separated from the outflowing purified water by sedimentation. Chemical coagulants are not used. Three main technologies are used in the investigated WWTPs (Fig. 1).

In the simplest process, implemented at WWTPs 1 and 7, the oxidation of organic matter by heterotrophic aerobic microorganisms and the oxidation of ammonium by nitrifiers with the formation of nitrate are carried out in plug-flow bioreactors under aerobic conditions. The sludge mixture moves continuously along an elongated corridor from the point of mixing of the return AS with incoming wastewater to the clarifier. Wastewater is supplied either only at the beginning of the bioreactor (Conventional Activated Sludge process, CAS) (WWTP7), or along the entire aeration tank corridor (Step Feed Process, SF) (WWTP1). The entire volume of the bioreactor is aerated by blowing air.

The nitrification/ denitrification technology (N-DN) is realized at WWTP2. Wastewater is fed into the plug-flow bioreactor at the beginning and in the middle of the bioreactor and passes through zones without aeration, each followed by an aerobic zone. In these two anoxic zones, denitrification occurs due to the organic matter contained in the wastewater and nitrates contained in the return AS mixture (in the first anoxic zone) or arriving from the first aerobic zone (in the second anoxic zone). In two aerated zones organics and ammonium are oxidized.

Six other WWTPs are operated by anaerobic/anoxic/oxic process, also known as the University of Cape Town (UCT) technology. At the first stage, the sludge mixture enters the anaerobic zone, where phosphate-accumulating microorganisms (PAO) consume easily degradable organic matter, then to the anoxic zone, where denitrification and accumulation of phosphates by denitrifying PAO occur, and finally to the aerobic zone, where organic matter and ammonium are oxidized. Recycling from the aerobic zone ensures the inflow of nitrates into the anoxic zone, and the recycle of the AS mixture from the end of anoxic zone into the anaerobic zone minimizes the ingress of nitrates. Plug-flow bioreactors are used at WWTPs 4, 8 and 9, and carousel bioreactors at WWTPs 3, 5 and 6 (Supplementary Fig. S1).

The bulk dissolved oxygen concentration in aerobic zones of all bioreactors was 2–3 mg/L, the solid retention time was 15–25 days, and the hydraulic retention time was 8–12 h. The temperature in the time of AS sampling was 18–20 °C, and did not fall below 16 °C even in winter. The concentrations of AS were similar (2–4 g/L) in all WWTPs, and volatile suspended solids accounted for 60–65%.

### Sampling and chemical analysis

The AS samples were obtained from nine wastewater treatment facilities at at Luberetsky and Kuryanovsky WWTP complexes in the Moscow region between March and May 2021. Samples of return activated sludge at 9 WWTPs (each in three replicas, 27 samples in total) were taken in BD Falcon tubes and immediately transferred to the laboratory at + 4 °C.

Samples of inflow and outflow water were collected between December 2020 and May 2021 and kindly provided by “Mosvodokanal” JSC. Chemical analysis was carried out according to standard methods. Water quality values (biochemical oxygen demand (BOD), chemical oxygen demand (COD), ammonium nitrogen (NH_4_-N) and phosphorus (P-PO_4_) of influent and effluent, as well as nitrate nitrogen (NO_3_-N) in the effluent were measured using environmental standard methods twice a week for 6 months. Monthly average values are shown in Table 1.

### DNA isolation, amplification and sequencing of the 16S rRNA gene fragments

Total genomic DNA from each AS sample was extracted using a Power Soil DNA isolation kit (MO BIO Laboratories, Inc., Carlsbad, CA, USA) and stored at − 20 °C. PCR amplification of 16S rRNA gene fragments comprising the V3–V4 variable regions was carried out using the universal prokaryotic primers PRK 341F (5′-CCTAYG GGDBGCWSCAG) and PRK 806R (5′-GGA CTA CNVGGG THTCTAAT)^25^. The PCR fragments were bar-coded using the Nextera XT Index Kit v.2 (Illumina, San Diego, CA, USA) and purified using Agencourt AMPure beads (Beckman Coulter, Brea, CA, USA). The concentrations of PCR products were determined using the Qubit dsDNA HS Assay Kit (Invitrogen, Carlsbad, CA, USA). All PCR fragments were then mixed and sequenced on Illumina MiSeq (2 × 300 nt from both ends). Pairwise overlapping reads were merged using FLASH v.1.2.11^26^. The final dataset consisted of 2,173,862 16S rRNA gene reads (Supplementary Table S1).

### Bioinformatics analysis of microbial community composition and diversity

All sequences were clustered into operational taxonomic units (OTUs) at 97% identity using the USEARCH v. 11 program^27^. Low quality reads, chimeric sequences, and singletons were removed by the USEARCH algorithm. To calculate OTU abundances, all reads obtained for a given sample (including singletons and low-quality reads) were mapped to OTU sequences at a 97% global identity threshold by USEARCH. The taxonomic assignment of OTUs was performed by searching against the SILVA v.138 rRNA sequence database using the VSEARCH v. 2.14.1 algorithm^28^. The recently developed MiDAS 4^14^, a full-length 16S rRNA gene reference database for wastewater treatment systems, was used to taxonomically identify OTUs up to the species level in the same way.

The diversity indices at a 97% OTU cut-off level were calculated using Usearch v.11^27^. To avoid sequencing depth bias, the number of reads generated for each sample were randomly sub-sampled to the size of the smallest dataset (94,942 reads) using the «single_rarefaction.py» script of QIIME^29^.

Calculation of Jaccard and weighted Unifrac distance metrics and trees was performed applying “beta_div” command in USEARCH. For the UniFrac analyses, a tree for the OTUs based on the sequence identity was constructed in USEARCH using “cluster_agg” command.

### Integration of data from this study with data from GWMC

All 16S rRNA gene sequences assigned to OTUs obtained in the present work were mapped to OTU sequences from GWMC (http://gwmc.ou.edu/). About 95% of sequences obtained in our experiments were mapped to GWMC OTUs at a 97% global identity threshold. OTU tables of both sets of samples were merged using command of “otutab_merge” of USEARCH. The obtained combined OTU table served as the input data for constructing a neighbor-joining tree generated from the Bray–Curtis dissimilarity matrix, which was calculated using the “beta_div” command of the Usearch program. The obtained tree was annotated and visualized in R package using ggtree method^30,31^.

### Network analysis

Co-occurrence networks were inferred based on a Spearman correlation matrix^32^ and constructed using only significant correlation^33^. The cutoff for correlation coefficients was determined to be 0.6 and the cutoff for adjusted *p*-values was 0.001^34^. Only OTUs, the relative abundance of which was at least 0.5% in at least one sample, were included in the analysis. Visualization of co-occurrence network was performed using Cytoscape v.3.8.2 platform^35,36^.

### Data availability

The raw data generated from 16S rRNA gene sequencing have been deposited in the NCBI Sequence Read Archive (SRA) and are available via the BioProject PRJNA764866.

## Results and discussion

### Performance characteristics of WWTPs

Three main technologies are used in the investigated WWTPs. The simplest aeration process is implemented at WWTPs 1 and 7, where wastewater is supplied either at the beginning of the bioreactor corridor (CAS process, WWTP7), or along the entire aeration tank (SF process, WWTP1). The WWTP1 removed more than 98% of organics (according to the BOD data) and about 90% of ammonium, while the purification efficiency of WWTP7 was lower (95% removal of organics and 70% of ammonium) (Table 1). Interestingly, although these units were not designed to remove phosphorus, the WWTP7 removed more than 70% of phosphorus, while in WWTP1 its removal was inefficient. The nitrification/ denitrification technology, realized at WWTP2, enabled removal of more than 98% of organics and more than 99% of ammonium, while phosphorous was not removed (Table 1). Six WWTPs operated by the UCT technology enabled efficient purification of the wastewater from both organics (> 98%), ammonium (> 99%) and phosphorous (> 90%) (Table 1). The concentrations of N-NO_3_ in the effluent were much lower than N-NH_4_ in the influent indicating efficient denitrification in all WWTPs (Table 1).

### Diversity of microbial communities of AS

**Figure 2:**
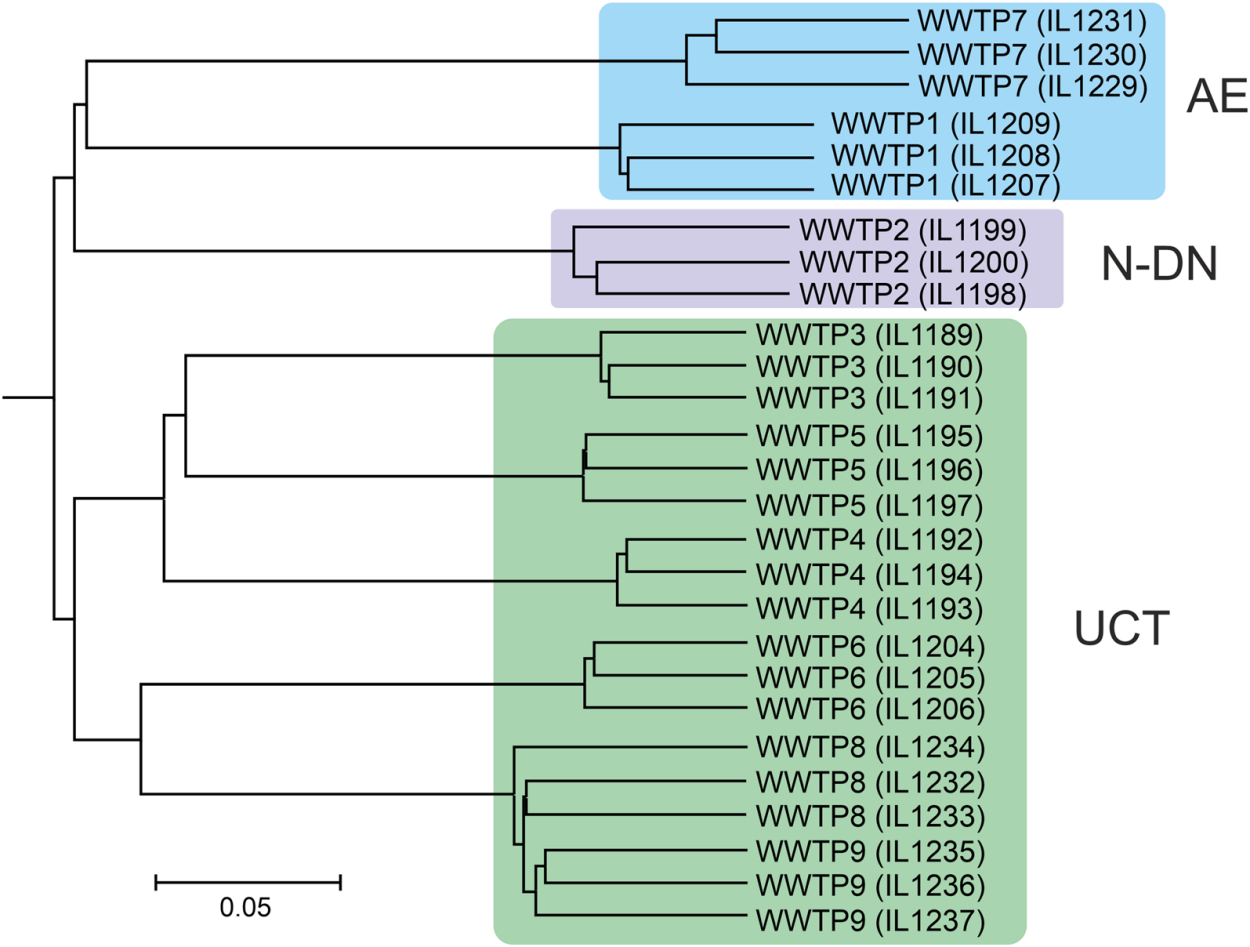
Neighbor joining tree illustrating weighted UniFrac distances between microbial communities of AS samples from 9 WWTPs (three replications). Sample IDs are shown in brackets after the WWTP number.

Between 23,167 and 84,634 16S rRNA gene sequences were obtained for 27 analysed AS samples (9 WWTPs, 3 replicas) and clustered into 14,690 OTUs at the level of 97% identity. Neighbour-joining trees based of the UniFrac analysis (Fig. 2) and Jaccard similarity (Supplementary Fig. S1) of OTU datasets revealed that replicate samples formed distinct branches for all WWTPs except for WWTP8 and WWTP9 which use identical bioreactors and treatment technologies. Therefore, for subsequent analysis, for each of the 9 WWTPs, three replicates were combined into one dataset. Both UniFrac and Jaccard trees revealed clustering of samples according to the technology used (Fig. 2 and Supplementary Fig. S2).

The number of species-level OTUs present in AS at individual WWTPs ranged between 3860 and 4868, these values are typical for large-scale wastewater treatment plants^10^. Overall, the diversity of microbial communities did not significantly vary between WWTPs employing different technologies, while the evenness of microbial communities in plants operated by the UCT process was slightly lower than in the others (Supplementary Table S2).

### Microbial community patterns at the phylum level

**Figure 3:**
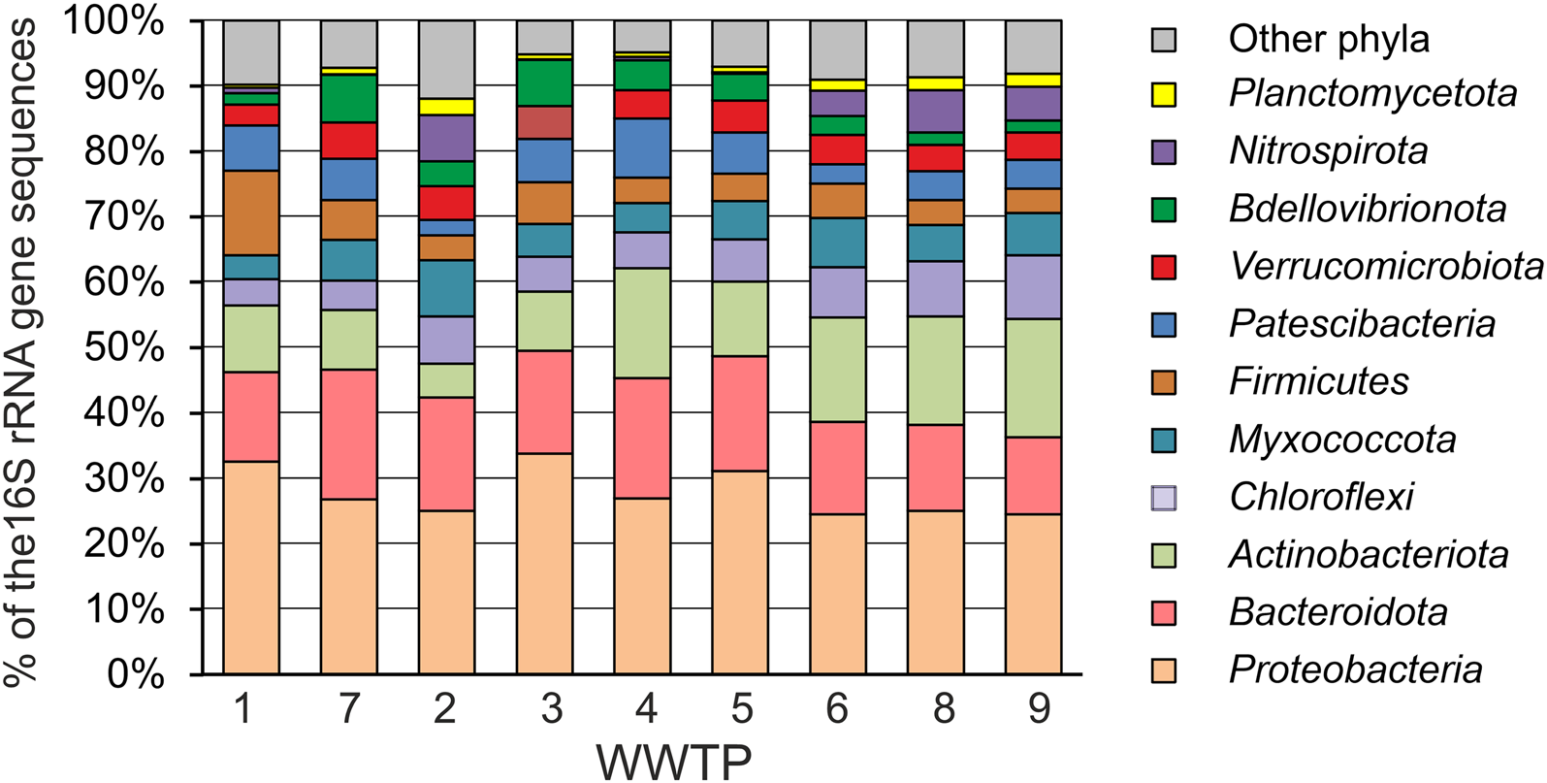
Bacterial and archaeal community composition in AS samples according to the results of 16S rRNA gene sequencing. The composition is displayed at the phylum level. Average values for three replicas are shown for each WWTP.

Taxonomic assignment of OTUs revealed the presence of 53 phylum-level lineages of Bacteria and Archaea, recognized in the Genome Taxonomy Database (GTDB)^37^. However, top 11 phyla comprising on average more than 1% of all the 16S rRNA gene sequences together accounted for more than 90% of the community (Fig. 3 and Supplementary Table S3).

Archaea represented less than 2% of sequences in all samples and were assigned to the phyla *Nanoarchaeota, Halobacterota, Euryarchaeota* and *Thermoplasmatota*; each was detected in all samples. Besides members of the phylum *Nanoarchaeota*, known to comprise partner-dependent parasites or symbionts with small genome size and limited metabolic capacities^38^, most of other Archaea represented known methanogenic lineages of the families *Methanobacteriaceae* and *Methanosaetaceae*.

Bacterial communities were dominated by the *Proteobacteria* (on average 27.8% of 16S rRNA gene reads), mostly of classes gamma (23.5%) and alpha (4.3%). Other major groups were the *Bacteroidota* (15.7%), *Actinobacteriota* (12.5%), *Chloroflexi* (6.6%), *Myxococcota* (5.9%), *Firmicutes* (5.6%), *Patescibacteria* (5.5%), *Verrucomicrobiota* (4.5%), *Bdellovibrionota* (3.9%), *Nitrospirota* (2.7%), and *Planctomycetota* (1.3%). The relative abundances of these lineages in different samples differ by no more than several times, with the exception of *Nitrospirota*, the share of which is minimal (0.07%) in WWTP3 and maximal (7.1%) in WWTP2. All other bacterial phyla accounted for less than 2% of 16S rRNA gene reads in all samples except for the *Campylobacterota* representing about 3.5% of the community in WWTP1.

### The main microbial drivers of wastewaters treatment

The biological wastewater treatment includes several microbial-driven processes, such as mineralization of organics by heterotrophs, oxidation of ammonia to nitrite and finally to nitrate, denitrificartion with the production of N_2_ gas, enhanced biological phosphorus removal etc. In this section we analyze the presence of the microbial groups which could be involved in these processes. The activities of microbial processes depend on the absolute concentrations of microorganisms, but given that the concentration of AS in all bioreactors was similar, we can consider the efficiency of processes in relation to the relative abundance of the corresponding functional group in the community. Data on the shares of the discussed groups of microorganisms are shown in Table 2.

**Table 2:** Relative abundancies (% of total 16S rRNA gene sequences) of microbial genera involved in nitrogen and phosphorous removal.

WWTP ID	WWTP1	WWTP2	WWTP3	WWTP4	WWTP5	WWTP6	WWTP7	WWTP8	WWTP9
Technology	SF	N-DN	UCT	UCT	UCT	UCT	CAS	UCT	UCT
**Ammonia oxidizers**									
*Nitrosomonas*	0.15	1.81	0.61	1.24	0.87	0.85	0.25	2.62	2.37
*Nitrosospira*	0.00	0.00	0.00	0.00	0.00	0.00	0.00	0.02	0.02
**Nitrite oxidizers**									
*Nitrospira*	0.78	7.06	0.07	0.48	0.20	3.92	0.08	6.49	5.20
*Ca*. Nitrotoga	0.00	0.00	0.26	0.46	0.38	0.01	0.01	0.00	0.00
**PAO (including potential)**									
*Ca*. Accumulibacter	0.59	0.12	1.01	0.43	0.76	0.48	0.90	0.73	1.05
*Tetrasphaera*	1.35	0.36	1.32	0.80	1.08	0.42	0.81	0.19	0.23
*Zoogloea*	0.66	0.19	0.35	0.09	0.23	0.48	1.41	0.15	0.13
*Thiothrix*	0.93	0.04	0.20	0.03	0.01	0.03	1.22	0.00	0.01
*Dechloromonas*	5.03	0.58	5.84	3.07	3.85	1.45	1.48	1.60	1.87
*Ca*. Obscuribacter	0.37	0.88	0.11	0.56	0.17	1.85	0.00	0.07	0.04
*Gemmatimonas*	0.01	0.03	0.01	0.00	0.00	0.03	0.06	0.03	0.03
*Thauera*	0.98	1.42	0.24	0.44	0.25	1.74	0.69	2.24	1.35
*Ca*. Microthrix	3.92	1.23	2.65	11.80	3.10	11.10	3.37	11.00	12.81
**GAO**									
Ca. Competibacter	3.54	0.98	8.50	7.13	7.33	2.51	0.95	2.53	3.66
*Defluviicoccus*	0.05	0.02	0.03	0.06	0.05	0.03	0.02	0.04	0.05

#### Oxidation of ammonia and denitrification

Oxidation of ammonia to nitrate via nitrite is usually accomplished by two groups of microorganisms, although complete oxidation of ammonia to nitrate by *Nitrospira* sp. (comammox process) has been also reported^39^. Among known ammonia oxidizers (AOM) only members of the family *Nitrosomonadaceae*, mostly of the genus *Nitrosomonas*, were detected. The relative abundance of *Nitrosomonadaceae* clearly correlated with efficiency of ammonia removal: it ranged from 0.8% to 2.9% in all WWTPs enabling good removal of ammonia (> 99%) and was below 0.4% in WWTPs 1 and 7 where ammonia removal was less efficient.

Two genera of nitrite-oxidizing bacteria (NOB) were identified, *Nitrospira* and *Candidatus* Nitrotoga. The relative abundance of *Nitrospirae* sp. was the highest (7.06%) in WWTP2, which uses nitrification–denitrification process and much lower in WWTPs 1 and 7 (0.78% and 0.08%). WWTPs using the UCT process in terms of *Nitrospirae* content were clearly divided into two groups. AS samples of WWTPs 6, 8 and 9 harbored between 3.9% and 6.5% of *Nitrospirae* sp., while its relative abundance was less than 0.5% in WWTPs 3, 4 and 5. However, microbial communities of these three bioreactors contained 0.26 to 0.46% of *Ca.* Nitrotoga, nearly absent in other WWTPs. This recently described *Ca*. Nitrotoga species can be functionally and sometimes dominant important nitrite oxidizers in WWTPs due to their relatively higher resistance to free nitrous acid and free ammonia than other NOB and the presence of complete pathways for hydrogen and sulfite oxidation, suggesting that alternative energy metabolisms enable these bacteria to survive nitrite depletion^40–42^.

Regardless of the used purification technology and the efficiency of ammonia removal, the concentration of N-NO_3_ in the effluent was in the range from 7 to 10.5 mg/L, which corresponds to 15–20% of N–NH_4_ in the influent and indicates effective denitrification and removal of nitrogen in the gaseous form. The presence of numerous heterotrophic denitrifying bacterial is consistent with this observation.

#### Biological phosphorus removal

The next main issue of wastewater treatment is the removal of phosphorus. This process is carried out by microorganisms of the group of phosphate-accumulating organisms (PAO) capable of intracellular accumulation of polyphosphates under cyclic growth conditions, with alternated presence and absence of electron acceptors^43–46^.

Typical PAO, most often found in WWTPs, are members of the candidate species *Candidatus* Accumulibacter phosphatis (family *Rhodocyclaceae*, *Gammaproteobacteria*)^47–50^. During the anaerobic phase, *Ca*. Accumulibacter phosphatis takes up volatile fatty acids present in the wastewater and stores the carbon from these substrates intracellularly as polyhydroxyalkanoates^51^. At the same time, intracellular polyphosphate is degraded to form ATP, releasing phosphate into the medium. During the aerobic phase, stored polyhydroxyalkanoates are used for energy production while phosphate is taken up from the medium and accumulated as polyphosphate. *Ca*. Accumulibacter phosphatis were found in all AS samples. Its relative abundance was the lowest (0.12%) in WWTP2 employing the nitrification/ denitrification process and poorly removing phosphorus. In other plants *Ca*. Accumulibacter phosphatis accounted for 0.43 to 1.05% of the communities and its share does not correlate with the phosphorus removal efficiency or the type of treatment process.

Several other bacteria, despite the difference in their metabolism from *Ca.* Accumulibacter phosphatis, are considered as likely PAO^46^, including members of *Proteobacteria* (*Dechloromonas*^52,53^, *Zooglea*^54,55^, *Thauera*^56^, *Thiothrix*^57^, *Ca*. Accumulimonas^58^, *Malikia*^59^), *Actinobacteria* (*Tetrasphaera*^60,61^, *Microlunatus*^62^, *Candidatus* Microthrix^63^), *Gemmatimonadetes* (*Gemmatimonas*^64^) and *Melainabacteria* (*Candidatus* Obscuribacter phosphatis^65^). Among these bacteria, members of the genera *Tetrasphaera, Zoogloea, Thiothrix, Dechloromonas, Ca.* Obscuribacter, *Gemmatimonas*, and *Ca.* Microthrix were detected.

The most abundant potential PAO, *Ca.* Microthrix sp., (average share 6.8%) was presented in all WWTPs and its share was higher in WWTPs employing the UCT process than in other types of bioreactors. Earlier it has been shown that *Candidatus* Microthrix parvicella contained of large polyphosphate granules and this microbial group might have been responsible for phosphorus removal during the sludge bulking period when *Ca*. Accumulibacter phosphatis was excluded from the system^63^. *Dechloromonas* sp., denitrifying bacteria capable of acetate uptake and polyphosphate storage, accounted for 1.5% to 5.8% of AS microbiomes in bioreactors operating under aerobic and UCT processes, while in WWTP2 it accounted for only 0.6%. Members of the genera *Zoogloea* and *Thiothrix*, aerobic bacteria capable of denitrification, often occur in ASs^57,66,67^. Both genera were more abundant in WWTPs employing aerobic process. *Ca.* Obscuribacter was most frequent in the WWTP6 (about 1.9%) and accounted for less than 1% in other samples. Members of the genus *Tetrasphaera*, known to be capable of aerobic polyphosphate accumulation under condition of assimilating glucose and/or amino acids anaerobically in advance^68^ were found in all WWTPs. Their relative abundance was minimal in WWTPs 8 and 9 (about 0.2%) and reached 1.35% in WWTP1. Although nitrate-reducing bacteria of the genus *Thauera* are primary known for their ability to perform anaerobic degradation of aromatic and other refractory compounds^69^, recently *Thauera* sp. strain SND5 was found to be a phosphate-accumulating organism^56^. The relative abundance of *Thauera* sp. ranged from 0.2 to 2.2% and was maximal in WWTP8.

Glycogen accumulating organisms (GAOs) can accumulate glycogen and polyhydroxyalkanoates inside cells, but do not have ability to intracellular accumulation of polyphosphates. They are commonly found together with PAO in EBPR bioreactors where their predominance may lead to EBPR failure due to the competition for carbon source with PAO^70^. Known GAO belongs to the *Gammaproteobacteria* (*Candidatus* Competibacter phosphatis) and *Alphaproteobacteria* (*Defluvicoccus* sp*.*)^71,72^. *Ca.* Competibacter accounted for up to 8.5% of the communities in UCT WWTPs, but were less numerous (< 1%) in WWTPs 2 and 7. The relative abundance of *Defluvicoccus* sp. was less than 0.1% in all samples. Thus, in WWTPs using the UCT process, a high relative abundance of GAO did not adversely affected the efficiency of phosphorus removal.

#### Microbial community composition and efficiency of wastewater purification

All five WWTPs using the UCT process ensured effective removal of not only organic matter, but also nitrogen and phosphorus, which is consistent with the high content of nitrifying and phosphate accumulating bacteria.

WWTP2, using the nitrification–denitrification process, effectively removed nitrogen, and the relative abundance of nitrifiers in this AS community was the highest. However, there was almost no phosphorus removal, and the share of PAO was the lowest. The low efficiency of phosphorus removal and the low abundance of PAO in WWTP2 are associated with the absence of anaerobic zones in bioreactors, as well as the consumption of easily degradable organic matter by denitrifying microorganisms.

Contrary to expectations, the two WWTPs, using a simple aeration process, provided the poorest removal of both organics and ammonia, while the nitrate content in the effluent was approximately the same as in other installations. However, the total nitrogen content in the form of nitrate and ammonium in the effluent was much lower than in the influent, indicating that nitrogen was removed during denitrification. Probably, under conditions of insufficient aeration, formation of local anoxic zones within microbial flocs facilitates the denitrification performed by heterotrophic bacteria^73^, while part of the ammonia and organic matter remain non-oxidized and enters the effluent water.

Interestingly, relatively efficient phosphorus removal was observed in WWTP7 but not in WWTP1. These two bioreactors differ in that in WWTP7 the organic-rich influent is fed to the beginning of the bioreactor, while in WWTP1 it is distributed along the entire length. It is likely that in WWTP7 local organic-rich anaerobic zones are formed within AS flocks near the point of influent supply, while in WWTP1 the concentration of organic substances is everywhere lower. In such organic-rich zones denitrifying PAO of the genera *Zoogloea* and *Thiothrix* can proliferate and their shares were maximal in WWTP7.

### Other abundant community members

Analysis of the compositions of microbial communities revealed 16 genus-level lineages, the average share of which across nine WWTPs exceeds 1% (Supplementary Table S4). In total, these 16 genera accounted for 28 to 51% (on average about 36%) of analyzed microbiomes. The most abundant genus, *Ca*. Microthrix, was the most numerous in AS samples of WWTPs 4, 6, 8 and 9 (11—13%) using the UCT process, while in other samples its relative abundance was much lower (1.2—3.4%). Besides its potential role in phosphorus removal, *Ca*. Microthrix is known as a slowly growing filamentous bacterium causing bulking and foaming in activated sludge systems thus seriously affecting the stable operation of WWTPs^74^. The growth of cultured species of this genus, *M. parvicella*, is susceptible to both environmental and operational parameters, such as temperature, oxygen concentration, type of substrate, electron acceptor, organic loading, and sludge retention time (reviewed in^75^). Probably, enrichment of *Ca*. Microthrix in the AS of the UCT WWTPs was associated with low oxygen concentrations in the anaerobic and anoxic zones of these bioreactors, favorable for the growth of these bacteria^7^. The dominant *Ca*. Microthrix phylotype was identified as *Ca* Microthrix subdominans, a species typically present along with *Ca*. M. parvicella, although usually in lower abundances^76^.

Besides *Ca*. Microthrix and above mentioned *Ca*. Competibacter, *Dechloromonas, Nitrospira, Nitrosomonas* and *Thauera,* the list of most abundant genera includes 3 cultured *(Trichococcus*, *Haliangium*, and *Chitinivorax*) and 7 uncultured lineages, defined in the Silva database as OM27_clade (*Bdellovibrionota, Bdellovibrionaceae*), *Candidatus* Nomurabacteria (*Patescibacteria*), AKYH767 (*Bacteroidota, Sphingobacteriales*), a member of the family *Moraxellaceae* (*Gammaproteobacteria*) defined as “Agitococcus lubricus group’, mle1-27 (*Myxococcota*), env.OPS_17 (*Bacteroidota, Sphingobacteriales*), and OLB12 (*Bacteroidota, Microscillaceae*).

Members of the genera *Trichococcus, Haliangium*, and *Chitinivorax* are often found in AS of wastewater treatment facilities. *Trichococcus* sp. are filamentous bacteria that can degrade a wide range of carbohydrates^77^, while *Chitinivorax* sp. are devoted to the hydrolysis of chitin^78^. Similarly to *Dechloromonas* sp., members of the *Haliangium* are active denitrifiers in the AS communities^79^.

All uncultured genus-level lineages were previously detected in bioreactors treating wastewater^80–83^, but their functional roles remain elusive. The most abundant uncultured OM27_clade, with an average share of about 3%, was hypothesized to be predatory bacteria^84^. Members of the candidate genus OLB12 are aerobic heterotrophs abundant in the anammox granules collected from partial-nitritation anammox reactor^85^. The only cultured member of “Agitococcus lubricus group’, *A. lubricus*, is a lipolytic aerobic heterotroph found in freshwater bodies^86^. The relative abundance of this lineage reached 4% in WWTP2, while it was below 0.2% in all AS samples currently described in the MiDAS database^14^.

### Network analysis

**Figure 4:**
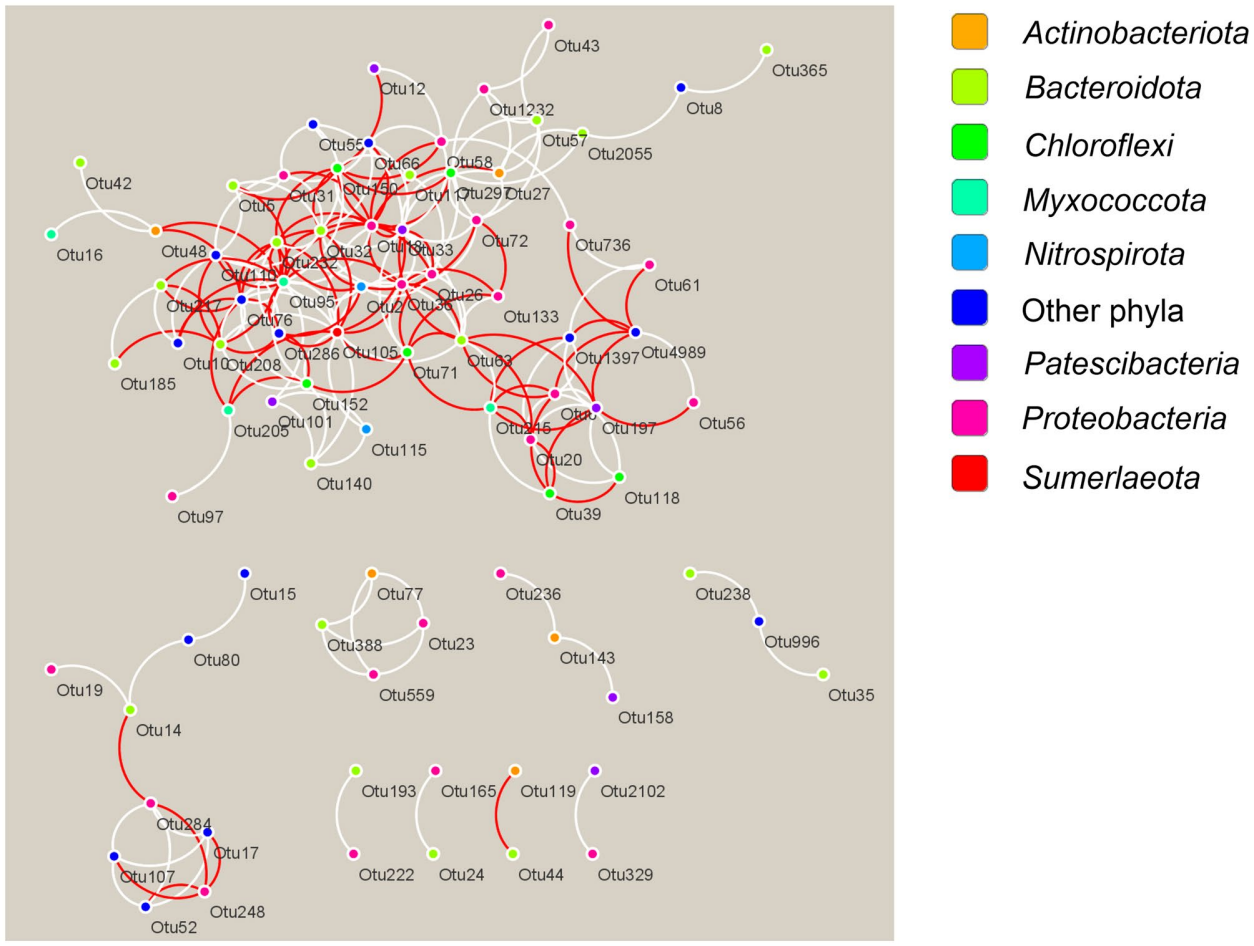
Network of co-occurring abundant microbial OTUs of AS based on correlation analysis. A connection stands for a strong (Spearman’s rho > 0.6) and significant (adjusted *p* value < 0.001) correlation. OTUs are colored according to phylum. Co-presence and mutual exclusion of OTUs are shown in white and red lines, respectively.

Various functional characteristics of microorganisms are closely related to the differentiation of ecological niches, and network analysis allows one to deduce patterns of interactions that develop within the WWTP ecosystem. Network analysis indicated possible cooperation (co-occurrence) and mutual exclusion among diverse microorganisms in WWTPs (Fig. 4).

Of 104 OTUs, with abundances more than 0.5% in at least one of the AS samples, 83 had statistically significant connections with other OTUs the maximum number of which reached 18 (Supplementary Table S5). OTUs with a high number of connections (“degree”) are considered as hub members, supposed to be key players in sustaining the overall community^87^. The largest number of connections (18) was observed for OTU assigned to the genus *Haliangium*, whose members, together with *Dechloromonas* sp., are active denitrifiers in WWTPs communities^79^. Notably, bacteria participating in ammonia and phosphorous removal were among the hub members, namely, *Nitrosomonas*, *Nitrospira*, *Ca*. Competibacter, *Dechloromonas,* and *Thauera*. Positive connection was detected between denitrifiers (*Thauera* Otu26 and *Haliangium* Otu95) and bacteria involved in the oxidation of ammonia (*Nitrosomonas* Otu18 and *Nitrospira* Otu2). This link is consistent with the fact that the source of nitrate is mainly microbial oxidation of ammonium, rather than influent sewage water. Interestingly, we did not detected mutual exclusion between *Ca*. Accumulibacter and *Ca*. Competibacter.

The nest largest number of connections was detected for Otu105, assigned to the candidate genus *Sumerlaea* (candidate phylum *Sumerlaeota*, previously known as BRC1). Members of this genus were predicted to be metabolically versatile bacteria capable to utilize various carbohydrates, including chitin, performing fermentation as well as complete oxidation of organic substrates through aerobic and anaerobic (with nitrate and sulfur compounds) respiration^88^. Co-presence of *Sumerlaea* and denitrifiers (*Thauera* and *Haliangium*) suggest that *Sumerlaea* sp. could be involved in degradation of organic substrates linked to dissimilatory nitrate reduction. Interestingly, no connection to other community members was found for the most abundant species, *Ca*. Microthrix (Otu1). *Ca*. Microthrix has been considered a specialized consumer of long-chain fatty acids that have been confirmed to be the key carbon and energy sources for its growth both under aerobic, anoxic and anaerobic conditions^75,89^. Probably due to such metabolic specialization *Ca*. Microthrix has limited interactions with other community members.

### Microbial communities of Moscow WWTPs on a global scale

Variations in community composition are key points for understanding community functioning. To understand how the composition of microbial communities of AS varied across different geographic locations, we compared taxonomic structures of our microbiomes with that reported in a worldwide study performed by a Global Water Microbiome Consortium^10^. That study showed that although the taxonomic community structures observed on different continents were not clearly separated at the OTU level in two dimensional ordinations, it was significantly different between any two continents^10^, and although the type of treatment process exerted significant effects on microbial community structures, it was overwhelmed by continental geographical separation^10^.

Despite its high diversity, AS microbiome has a small global core bacterial community (28 OTUs) that is strongly linked to WWTP performance and accounted for an average 12.4% of the 16S rRNA gene sequences in AS samples^10^. 26 of these OTUs were present in Moscow WWTPs and accumulated from 11.3 to 23.5% of 16S rRNA gene reads (Supplementary Table S6).

**Figure 5:**
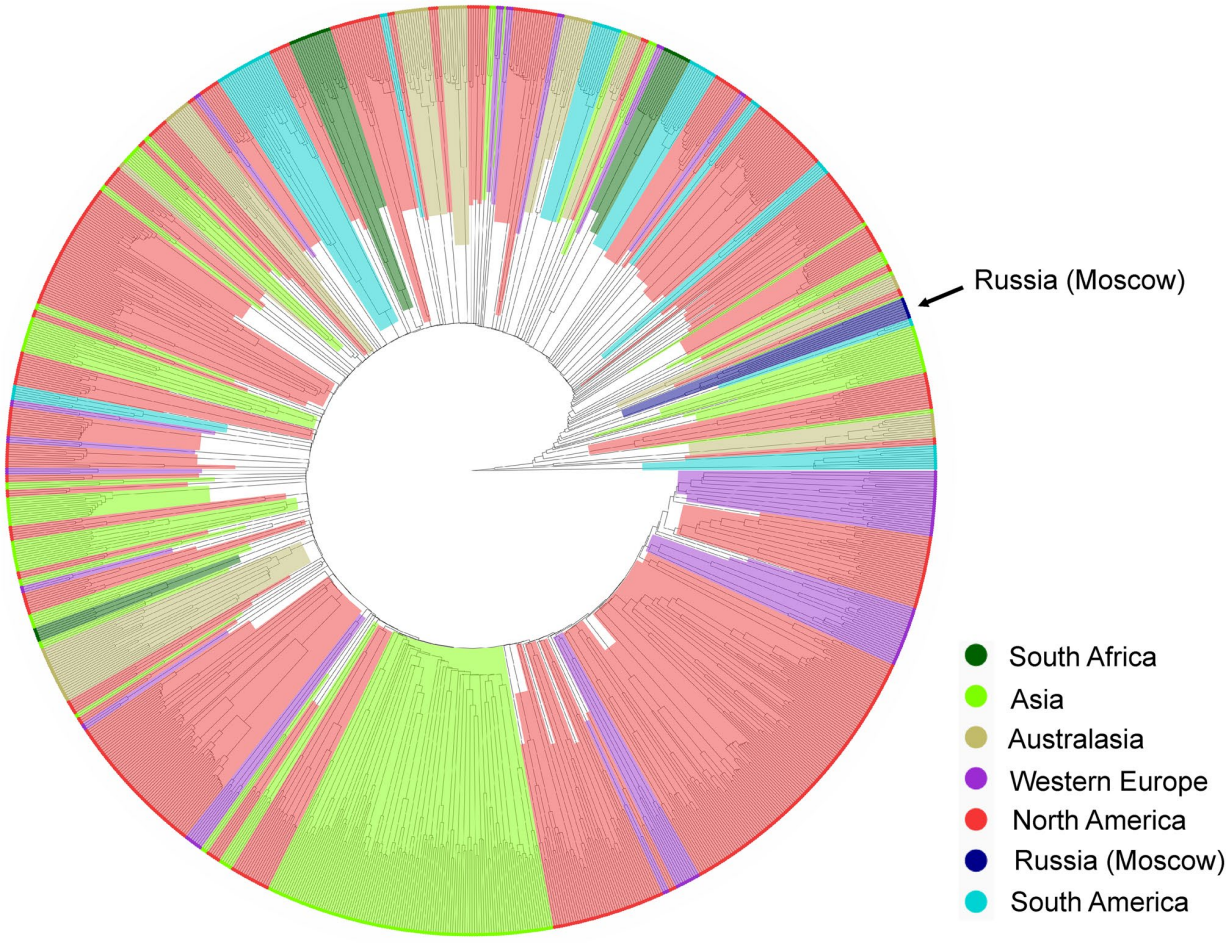
Neighbor joining tree illustrating clustering based on the Bray–Curtis dissimilarity between microbial communities of AS samples from 9 WWTPs obtained in this study and ones reported by a Global Water Microbiome Consortium (http://gwmc.ou.edu/). Samples are colored according to geographic location.

Clustering based on the Bray–Curtis dissimilarity showed that all samples from Moscow WWTPs clustered together and formed a distinct branch not embedded into European or any other large groups, but forming a sister lineage to a subsets of Asian, South American and North American clusters (Fig. 5). These data indicate that influent characteristics, related to cultural, social and environmental factors in each region, could be more important than a plant operating conditions. Similar observations have been previously reported for the microbial communities of AS from WWTPs in Korea and Vietnam^2^. For example, due to the relatively low cost of water for household consumption, wastewater in Moscow has a relatively low content of organic matter compared to the values typical for most large cities (COD of 600–900 mg/L). It should be noted that the primers used in our work for the 16S rRNA gene profiling differed from those used by the GWMC consortium (515F/806R), which may affect the observed shares of particular microbial taxa. However, both primer pairs (515F/806R and 341F/806R) enabled good coverage of overall microbial diversity and provided similar estimates of the relative abundancies of most species in various environments^90,91^.

## Conclusions

This study provides the data on the composition of microbial communities of AS from large-scale WWTPs of the Moscow city and the evidences of the impact of purification technology. Although the taxonomic structure of communities at high ranks was similar and the most numerous groups were found in all WWTPs, UniFrac and Jaccard trees revealed clustering of samples according to the employed technology. WWTPs employing the UCT process enabled efficient removal of organics, nitrogen and phosphorus, while WWTPs operated under simple aeration or nitrification–denitrification process removed these components less efficiently. Co-occurrence network analysis provided information on key hub microorganisms in AS, which may be targeted for manipulating the AS stability and performance. Comparison of AS communities from Moscow WWTPs with ones analyzed worldwide by GWMC revealed that all Moscow samples clustered together, indicating that influent characteristics, related to cultural, social and environmental factors in each region, could be more important than a plant operating conditions.

## Supplementary Information

Supplementary Figure S1.

Supplementary Figure S2.

Supplementary Table S1.

Supplementary Table S2.

Supplementary Table S3.

Supplementary Table S4.

Supplementary Table S5.

Supplementary Table S6.
