# Supplementary material for: The structure of microbial communities of activated sludge of large-scale wastewater treatment plants in the city of Moscow
Source: Sci Rep. 2022 Mar 2;12:3458. doi: 10.1038/s41598-022-07132-4 (PMC8891259; doi:10.1038/s41598-022-07132-4)
Supplement: Supplementary file 1 — Supplementary Figure S1. [file 41598_2022_7132_MOESM1_ESM.pdf]

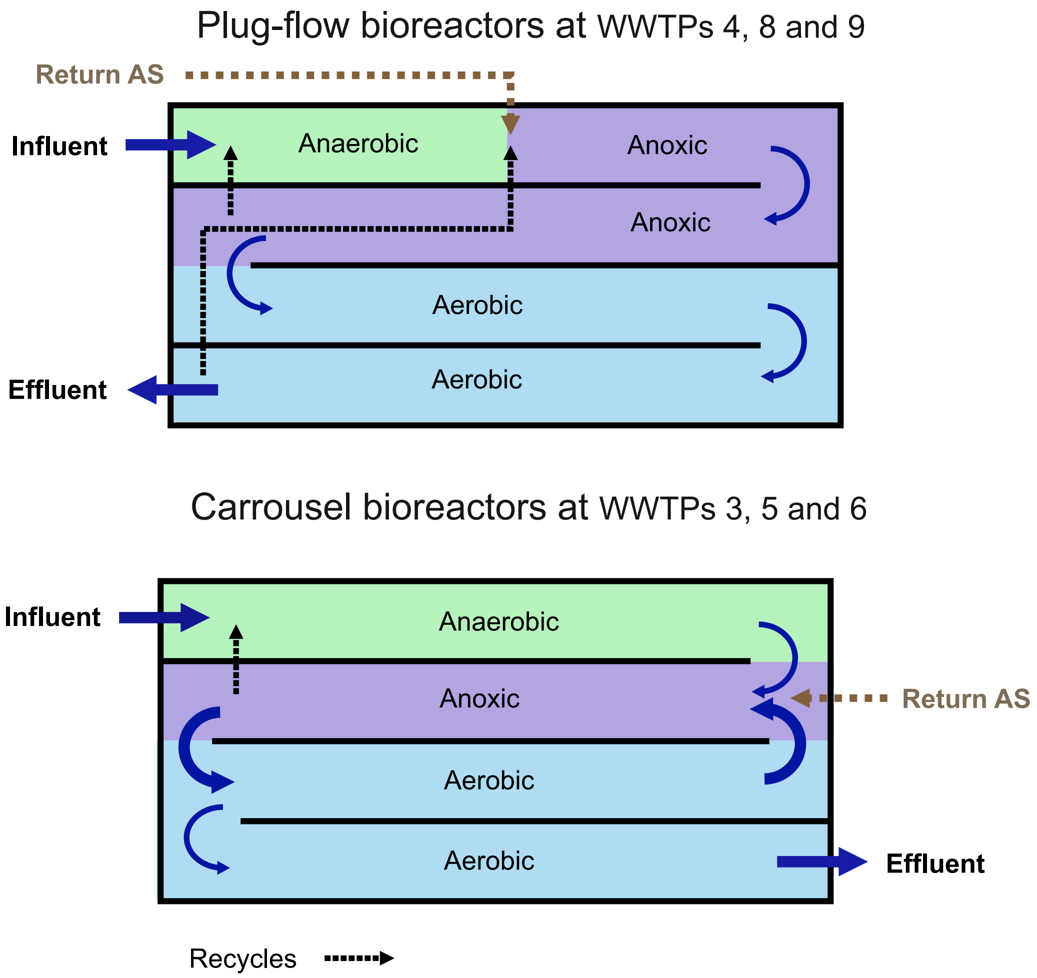

Supplementary Figure S1. Schemes of two types of bioreactors operated using the UCT technology.
