# Supplementary material for: The structure of microbial communities of activated sludge of large-scale wastewater treatment plants in the city of Moscow
Source: Sci Rep. 2022 Mar 2;12:3458. doi: 10.1038/s41598-022-07132-4 (PMC8891259; doi:10.1038/s41598-022-07132-4)
Supplement: Supplementary file 2 — Supplementary Figure S2. [file 41598_2022_7132_MOESM2_ESM.pdf]

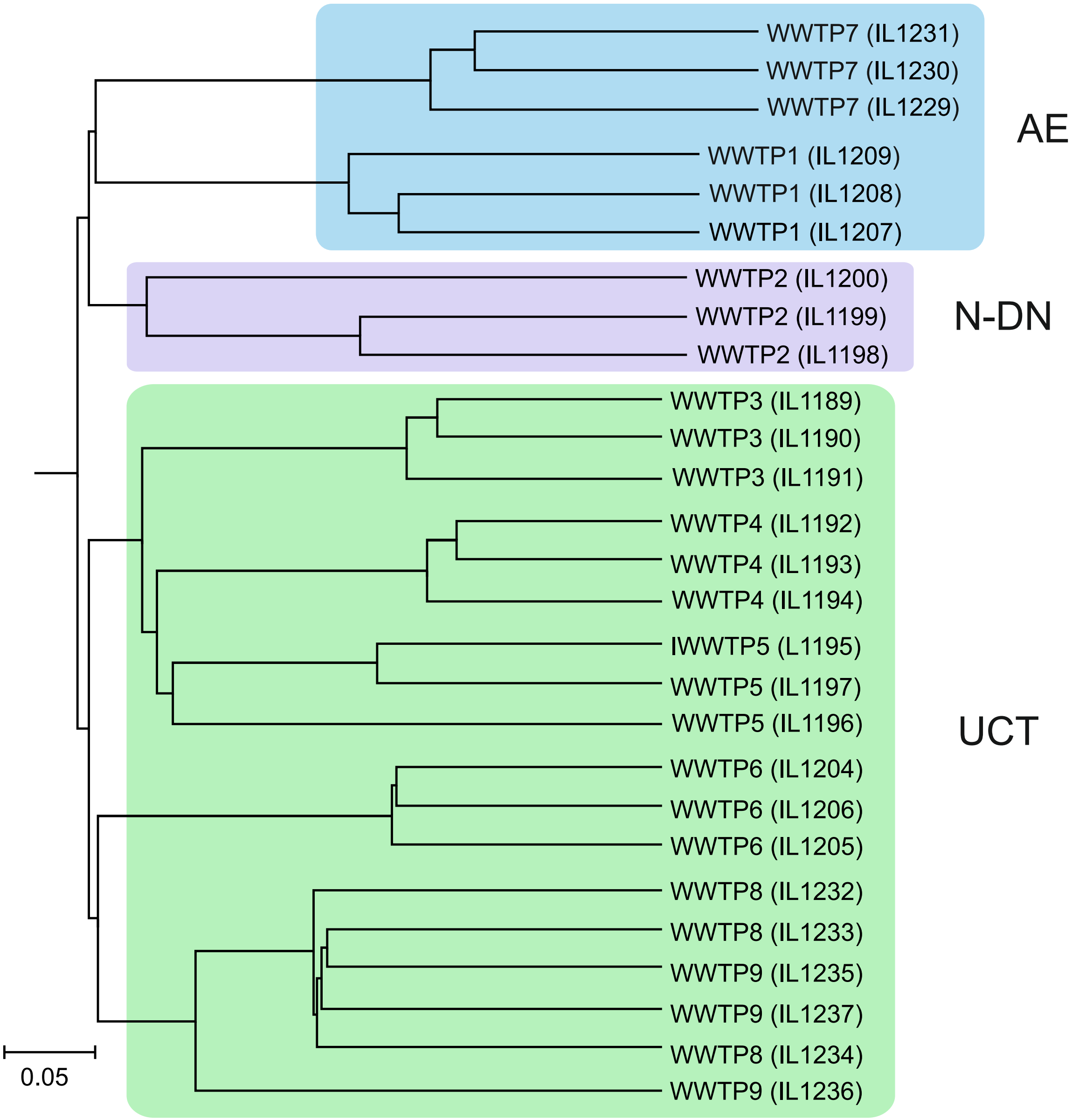

Supplementary Figure S2. Neighbor joining tree illustrating Jaccard distances between microbial communities of AS samples from 9 WWTPs (three replications). Sample IDs are shown in brackets after the WWTP number.
